# Clinical outcomes of septic patients according to the elapsed time before transfer to the intensive care unit

**DOI:** 10.1186/2197-425X-3-S1-A229

**Published:** 2015-10-01

**Authors:** A Gonçalves Dias, E Ribeiro dos Santos, D Faria Moura Junior, C Takao Lopes, B Murata Murakami

**Affiliations:** Hospital Israelita Albert Einstein, São Paulo, Brazil; Universidade Federal de São Paulo, São Paulo, Brazil

## Intr

Rapid response teams (RRT) are important systems for identifying patients requiring intensive car e[1]. Compliance with the process of care in sepsis can be increased by the activation of RRT, thereby reducing hospital mortality rate [2].

## Objectives

To investigate the correlation between the clinical outcomes of septic patients and the elapsed time before transfer to the intensive care unit (ICU).

## Methods

A retrospective descriptive study performed in a large hospital in São Paulo, Brazil, with all patients admitted to the ICU by RRT activation due to suspected sepsis, sepsis, severe sepsis or septic shock from January to December 2011.

## Results

39 patients were attended by RRT 5 to 20 minutes after activation. Thirty patients (76.9%) were immediately transferred to the ICU. The elapsed time since assessment to transfer to the ICU ranged from 15 to 30 minutes (n = 5, 12.9%), 30 minutes to 1 hour (n = 15; 38.5%), 1 to 2 hours (n = 8, 20.5%) and ≥3 hours (n = 8, 20.5%). As for the clinical outcome in the ICU, 20 (51.3%) had a clinical improvement, 14 (35.9%) died and 5 (12.8%) had an initial clinical deterioration with subsequent improvement. Clinical improvement or initial deterioration with subsequent recovery occurred mainly among patients transferred to the ICU within 15 minutes to 3 hours. In patients transferred after 3 hours, death was the most frequent outcome.

## Conclusions

The clinical outcomes of septic patients early transferred to the ICU are better than the outcomes of patients transferred later.
